# Altered spontaneous cortical activity predicts pain perception in individuals with cerebral palsy

**DOI:** 10.1093/braincomms/fcac087

**Published:** 2022-04-04

**Authors:** Michael P. Trevarrow, Anna Reelfs, Lauren R. Ott, Samantha H. Penhale, Brandon J. Lew, Jessica Goeller, Tony W. Wilson, Max J. Kurz

**Affiliations:** 1 Institute for Human Neuroscience, Boys Town National Research Hospital, Omaha, NE 68010, USA; 2 Department of Anesthesiology, University of Nebraska Medical Center, Omaha, NE 68198, USA; 3 Department of Pharmacology & Neuroscience, Creighton University, Omaha, NE 68178, USA

**Keywords:** magnetoencephalography, resting state, somatosensory, SII

## Abstract

Cerebral palsy is the most common paediatric neurological disorder and results in extensive impairment to the sensorimotor system. However, these individuals also experience increased pain perception, resulting in decreased quality of life. In the present study, we utilized magnetoencephalographic brain imaging to examine whether alterations in spontaneous neural activity predict the level of pain experienced in a cohort of 38 individuals with spastic diplegic cerebral palsy and 67 neurotypical controls. Participants completed 5 min of an eyes closed resting-state paradigm while undergoing a magnetoencephalography recording. The magnetoencephalographic data were then source imaged, and the power within the delta (2–4 Hz), theta (5–7 Hz), alpha (8–12 Hz), beta (15–29 Hz), low gamma (30–59 Hz) and high gamma (60–90 Hz) frequency bands were computed. The resulting power spectral density maps were analysed vertex-wise to identify differences in spontaneous activity between groups. Our findings indicated that spontaneous cortical activity was altered in the participants with cerebral palsy in the delta, alpha, beta, low gamma and high gamma bands across the occipital, frontal and secondary somatosensory cortical areas (all *p*_FWE_ < 0.05). Furthermore, we also found that the altered beta band spontaneous activity in the secondary somatosensory cortices predicted heightened pain perception in the individuals with cerebral palsy (*P* = 0.039). Overall, these results demonstrate that spontaneous cortical activity within individuals with cerebral palsy is altered in comparison to their neurotypical peers and may predict increased pain perception in this patient population. Potentially, changes in spontaneous resting-state activity may be utilized to measure the effectiveness of current treatment approaches that are directed at reducing the pain experienced by individuals with cerebral palsy.

## Teaser

Trevarrow *et al*. report alterations in spontaneous cortical activity across multiple spectral ranges in individuals with cerebral palsy. Interestingly, altered spontaneous activity in the beta range was correlated with increased pain perception in the secondary somatosensory cortices. These findings provide a new understanding of the neurological correlates of pain in cerebral palsy.

## Graphical Abstract

**Graphical Abstract:**
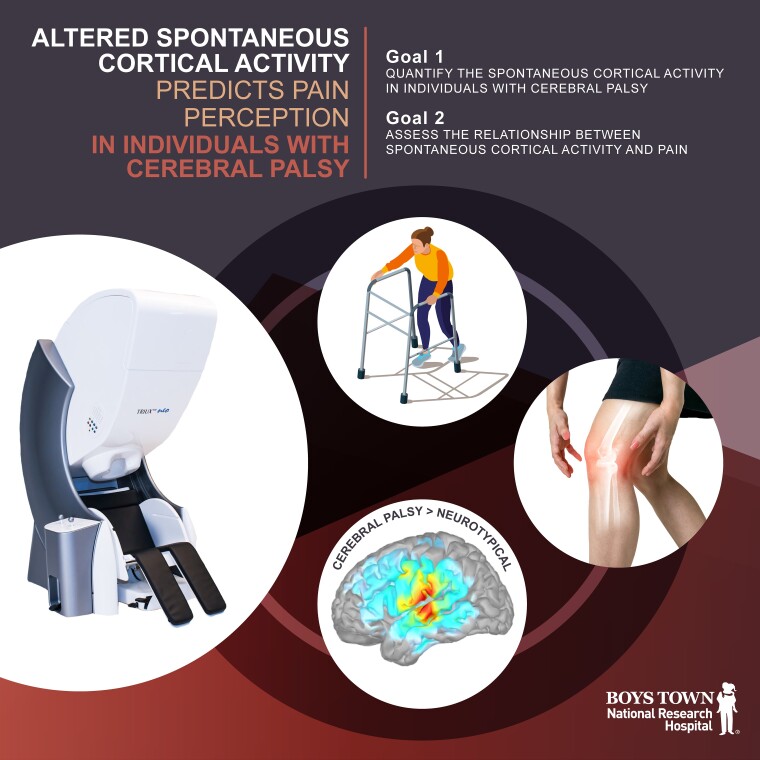

## Introduction

Cerebral palsy (CP) refers to a group of movement disorders that result from an insult to the developing brain, and it is one of the most prevalent neurodevelopmental disorders in the United States.^1^ In addition to the inherent deficits in motor function, these individuals also experience discrepancies in other major domains, including somatosensory^2–7^ and visual processing.^8–11^ Thus, the early brain damage in CP impacts multiple functions that extend beyond the motor system. Furthermore, individuals with CP often report increases in their daily pain perception, which can result in difficulty completing tasks of daily living, decreased sleep quality and reduced participation in peer-related activities.^12–17^ Consequently, pain poses a serious concern in this population and significantly reduces their quality of life. The specific neurological underpinnings that may contribute to the altered pain perception are unknown, and thus occupy a prominent knowledge gap in our ability to treat pain in those with CP.

The basis for the increased pain perception in individuals with CP is known to be multifaceted. The pain is primarily experienced in the lower back and lower extremities, resulting from several factors within both the musculoskeletal and nervous systems. Neuromuscular abnormalities, such as spasticity and dystonia, can contribute to intensified pain,^13,18^ but the pain may also be care-related and emerge after therapeutic or surgical interventions.^13,19,20^ Greater pain could likewise be a consequence of direct or indirect inflammatory factors^21,22^ or from poor bone mineral density and orthopaedic-related impairments.^23^ Pain perception tends to be more severe in individuals with CP that have emotional and psychosocial difficulties^14,24^ and higher gross motor function classification system (GMFCS) levels,^25^ and it tends to worsen with age. The latter may be due to joint contractures and secondary pathologies such as neuropathy and osteoarthritis prompting chronic pain.^21,25,26^ An alternative perspective is that the processing of pain may be primarily disrupted at the level of the cortex, in which there are both sensory and emotional processing networks within the primary somatosensory, secondary somatosensory, insular and cingulate cortices that work together for pain perception.^27,28^ Although this later premise is plausible, studies linking disruptions in cortical activity to the altered pain perceptions in individuals with CP have yet to be conducted.

Neuroimaging studies of resting-state brain activity have rapidly expanded over the past decade, as this methodology has been shown to provide unique insight into disruptions in the neural mechanisms involved in cognitive and sensorimotor function.^29–31^ Resting-state paradigms are beneficial because they evade the confounding effects of motivation and effort that can occur within task-based cognitive paradigms,^32^ making it ideal for studying individuals across a variety of patient populations. In fact, several studies have utilized functional (MRI) to investigate resting-state functional connectivity (FC) in individuals with CP.^33–37^ These studies have broadly shown that individuals with a spastic diplegic presentation tend to have increased and expanded FC in the sensorimotor cortices and the supplementary motor areas in comparison to their neurotypical (NT) peers, which is potentially a result of reorganization within the homuncular topology.^33,34^ Conversely, decreased FC has been found between the bilateral sensorimotor cortices, as well as the sensorimotor cortex and the cerebellum, cingulate motor area, visual cortices and parietal cortices in both spastic and dyskinetic CP.^33,36–39^ For example, our recent fMRI experimental work demonstrated decreased FC between motor and visual networks and connected these aberrations to altered gait kinematics.^40^ Thus, resting-state paradigms provide a viable approach to assessing how the initial brain insult alters cortical networks and has cascading effects on the overall neurophysiology of CP.

Recently, there has been growing interest in studying other neural parameters of resting-state, such as spontaneous cortical activity. Briefly, it is well known that human cortical neurons exhibit spontaneous firing in the absence of incoming exogenous and endogenous input. Across a neuronal population, these discharges summate with local dendritic currents and synaptic potentials to produce cortical rhythmic activity, which is often referred to as ‘spontaneous activity’. Previous literature has identified that spontaneous activity is aberrant in patient populations,^41–44^ and there are well-documented age-related changes in neural power within specific frequency bands.^45–49^ Furthermore, spontaneous cortical activity has previously been linked with alterations in oscillatory brain responses.^50–54^ We propose that quantifying spontaneous cortical activity may provide insight into the underlying factors driving the altered oscillatory responses seen within individuals with CP^55–63^ and further enhance the field’s insight into the neurological parameters that are fundamentally impacted by the perinatal brain insults associated with CP. Furthermore, increased spontaneous cortical activity within the beta band has previously been illustrated in patients with chronic pain,^64–68^ and when measured using magnetoencephalographic (MEG) imaging has been shown to be highly stable over at least a three-year period.^69^ The latter findings are highly important for any potential neural marker. Thus, a potential link may exist between disruptions in the brain’s intrinsic spontaneous cortical activity and the increased pain perception experienced by individuals with CP.

The overall goal of this investigation was to determine whether spontaneous cortical oscillations differ in individuals with CP compared to NT controls, and to identify whether such alterations in spontaneous oscillations were associated with the increased pain perception extensively documented in this population. To this end, we used MEG imaging to quantify the resting-state spontaneous cortical dynamics across the respective canonical frequency bands. We hypothesized that participants with CP would exhibit altered spontaneous cortical oscillations relative NT controls. Furthermore, we tested the premise that the processing of pain may be disrupted at the level of the cortex by assessing the relationship between the strength of spontaneous oscillations and the pain perceptions reported by participants with CP.

## Methods

### Participants

A cohort of 38 individuals with spastic diplegic CP and GMFCS levels between I and IV (Age = 22.08 ± 10.46 yrs., Females = 20) participated in this study. Individuals with GMFCS levels of I and II typically ambulate independently, although with slowed gait speed and atypical gait patterns. Individuals with GMFCS level of III often use assistive devices to ambulate, such as crutches, ankle-foot orthoses or wheelchairs. Individuals with GMFCS levels IV and V often use powered mobility devices. Additionally, we enrolled a cohort of 67 NT controls (Age = 19.56 ± 10.25 yrs., Females = 27). An independent samples *t*-test revealed that the two groups did not statistically differ by age (*P* = 0.231), and a chi-squared test revealed that the groups did not differ by sex (*χ*^2^ = 1.49, *P* = 0.222). The participants with CP had not undergone surgery within the last 6 months and had not had botulinum toxin injections in the past year. Parents of the child participants signed informed consent forms, and child participants signed assent forms before proceeding with the study. The Institutional Review Board reviewed and approved this study, and all protocols were in accordance with the Declaration of Helsinki.

### MEG data acquisition

These methods were performed according to Ott *et al*.^49^

### Structural MRI processing and MEG-MRI coregistration

Each participant’s MEG data was co-registered with the MNI 152 template MRI prior to source space analyses using Brainstorm,^70^ with the template MRI volume scaled to fit the scalp surface points when necessary. For a full description of these methods, refer to Ott *et al*.^49^

### MEG data pre-processing

Each MEG dataset was individually corrected for head motion and subjected to noise reduction using the signal space separation method with a temporal extension (tSSS; MaxFilter v2.2; correlation limit: 0.950; correlation window duration: 6 s^71^). MEG data processing was completed in Brainstorm^70^ and largely followed the analysis procedure outlined in.^72^ A high-pass filter of 0.3 Hz and notch filters at 60 Hz and at its harmonics were applied. Cardiac artefacts were identified in the raw MEG data and removed using an adaptive signal-space projection (SSP) approach, which was subsequently accounted for during source reconstruction.^73^ Data were then divided into four-second epochs for detection and rejection of bad segments of data based on amplitude and gradient distributions per participant.

### MEG source imaging and frequency power maps

As in Niso *et al*.,^72^ we computed minimum norm estimates normalized by a dynamic statistical parametric mapping (dSPM) algorithm for source imaging. To account for environmental noise, we utilized empty room recordings to compute a noise covariance matrix for source imaging.^74^ The forward model was computed using an overlapping spheres head model.^75^ Finally, the imaging kernel of depth-weighted dSPM constrained to the individual cortical surface^76^ was computed.

Using these source estimates, we then computed the power of cortical activity in canonical frequency bands: delta (2–4 Hz), theta (5–7 Hz), alpha (8–12 Hz), beta (15–29 Hz), low gamma (30–59 Hz) and high gamma (60–90 Hz). We used Welch’s method for estimating power spectrum densities (PSD) on each four-second epoch for each MEG recording, with one second sliding Hamming windows overlapping at 50%. We then standardized the PSD values at each frequency bin to the total power across the frequency spectrum. For each participant, we then averaged PSD maps across epochs to obtain one set of PSD maps per participant. Finally, we projected these maps onto the MNI ICBM152 brain template when scaling was used during coregistration^77^ and applied a 3 mm full width half max (FWHM) smoothing kernel. Ultimately, it was these normalized source maps per frequency band that were used for further statistical analysis.

### Pain surveys

A subcohort of the participants with CP (*N* = 22, Females = 12) that took part in an exploratory phase of the study aimed at assessing neurological components of pain completed the PROMIS Pediatric Pain Interference Scale.^78^ The scale consists of 20 questions that assess how pain has impacted activities of daily living within the past 7 days. The score on each question is ranked one to five, with one indicating that that facet of life has not been affected at all by pain, and five indicating that facet of life has been significantly affected by pain. The final score is a summation of all questions, with 100 indicative of the highest pain interference.

### Statistical analyses

We then analysed the whole-brain PSD maps in SPM12 to examine for spatially specific effects of group (CP versus NT). For each frequency band, we ran a mass univariate approach based on the general linear model to compare the spontaneous power between groups. To correct for multiple comparisons, we applied threshold-free cluster enhancement (TFCE^79^) with a weighting factor of *E* = 0.6 and a cluster level family-wise error (FWE) of 0.05 to the resulting statistical maps. Next, F-maps were thresholded utilizing the clusters that survived correction. Data from peak voxels were used to display the corresponding effects. Subsequently, we performed a hierarchical regression, in which we regressed the scores from the Pain Interference Scales on the spontaneous cortical activity from the peak voxel in the respective frequency bands. Next, GMFCS level was added to the regression model as a predictor to assess whether severity of impairment was related to pain interference. Finally, the interaction between GMFCS level and spontaneous cortical activity was added to the model to assess whether the severity of impairment modulated the relationship between spontaneous cortical activity and pain interference.

### Data availability

The data are available upon reasonable request to the corresponding author.

## Results

### Spontaneous neural dynamics

**Figure 1:**
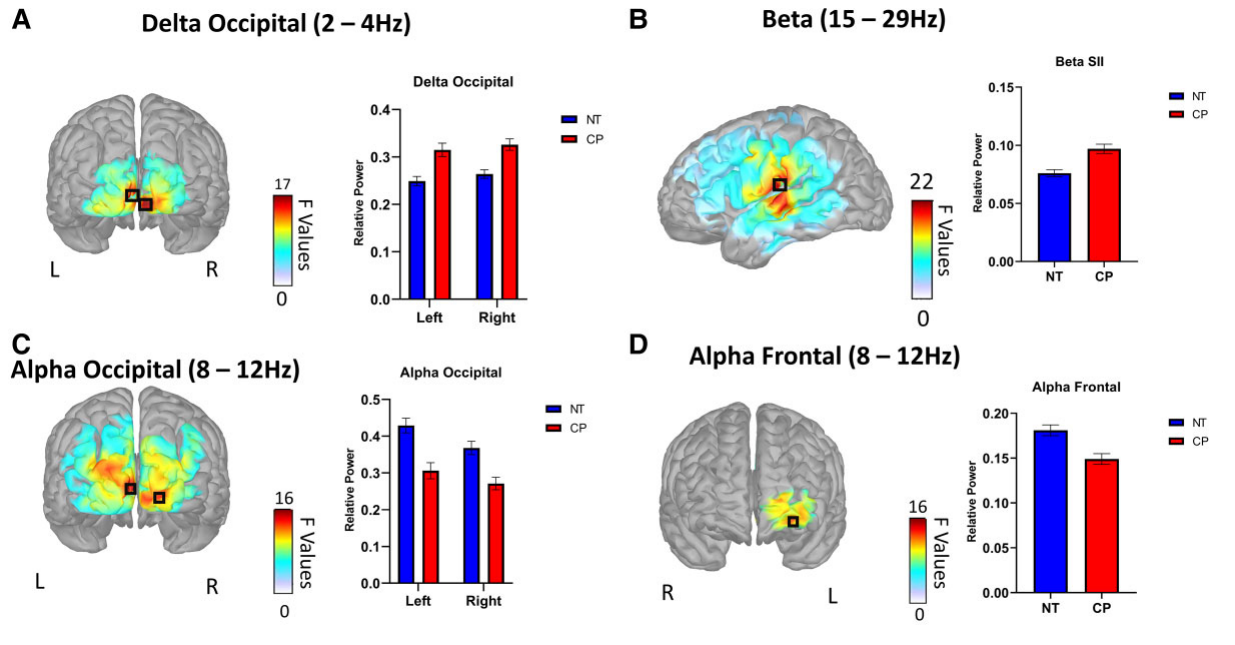
**Group differences in spontaneous cortical activity in the delta, alpha and beta bands**. Main effects of group: F-maps thresholded with threshold-free cluster enhancement (TFCE) depicting regions in the brain with group differences within the delta, alpha and beta bands, with a black box indicating the peaks. For display purposes, the corresponding bar graphs illustrate the group averages at the respective group difference peaks. (**A**) Delta (2–4 Hz) band power was higher in the individuals with CP in the left and right occipital cortices. (**B**) Beta (15–29 Hz) band power was higher in the individuals with CP in the left SII. Alpha (8–12 Hz) band power was decreased in the individuals with CP in the left and right occipital cortices (**C**) as well as the prefrontal cortex (**D**). NT represents neurotypical controls, while CP represents cerebral palsy.

**Figure 2:**
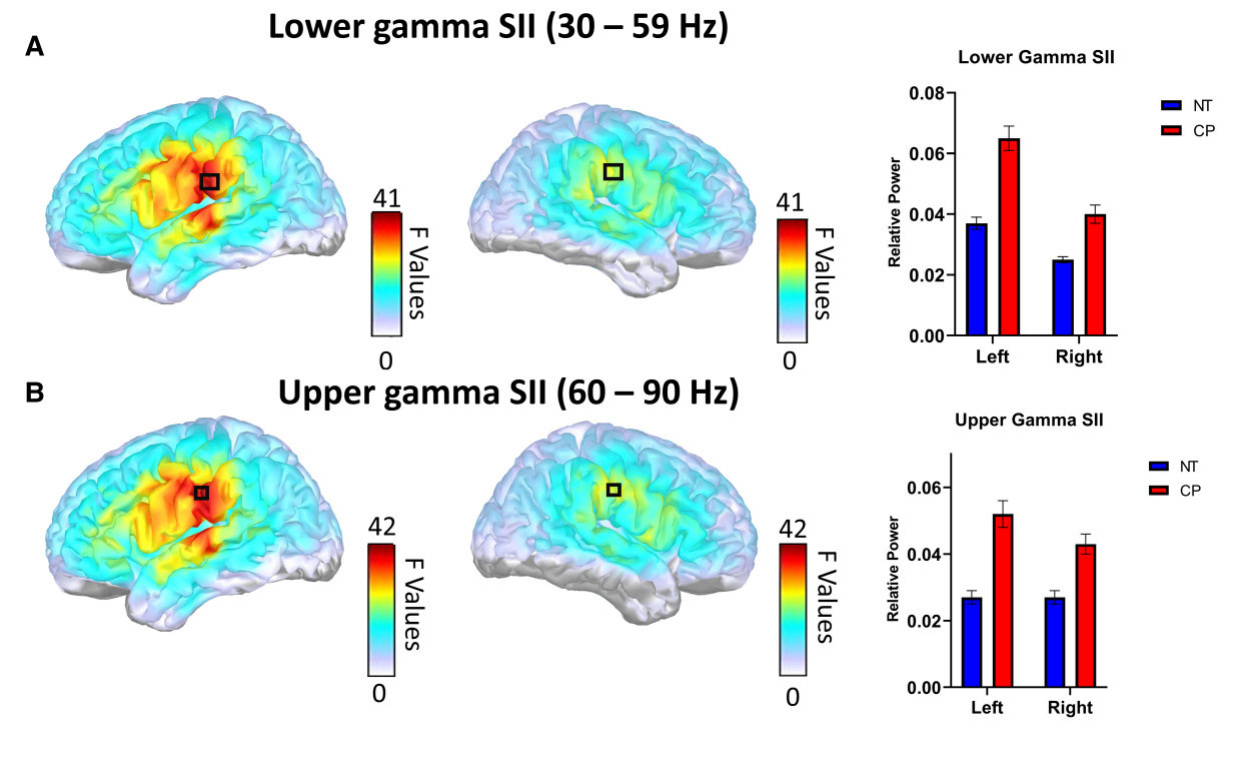
**Group differences in spontaneous cortical activity in the gamma band**. Main effects of group: F-maps thresholded with threshold-free cluster enhancement (TFCE) depict regions in the brain with group differences within the lower (**A**) and upper (**B**) gamma bands, with a black box indicating the peaks. For display purposes, the corresponding bar graphs illustrate the group averages at the respective group difference peaks. Lower (30–59 Hz) (top) and upper (60–90 Hz) (bottom) gamma band power were higher in the individuals with cerebral palsy within the left and right SII. NT represents neurotypical controls, while CP represents cerebral palsy.

In the delta band, the individuals with CP had greater power in the left (CP = 0.315 ± 0.083, NT = 0.249 ± 0.083, *p_FWE_* = 0.028) and right occipital (CP = 0.326 ± 0.075, NT = 0.264 ± 0.077, *p_FWE_* = 0.030) areas; Fig. 1). Within the alpha band, the individuals with CP had reduced power within the left (CP = 0.306 ± 0.022, NT = 0.429 ± 0.020, *p_FWE_* = 0.019) and right occipital (CP = 0.271 ± 0.018, NT = 0.368 ± 0.017, *p_FWE_* = 0.022) areas, as well as the left prefrontal cortex (CP = 0.149 ± 0.006, NT = 0.181 ± 0.006, *p_FWE_* = 0.029; Fig. 1). Within the beta band, the individuals with CP had greater power in the left secondary somatosensory cortex (SII) (CP = 0.097 ± 0.023, NT = 0.076 ± 0.022, *p_FWE_* = 0.015; Fig. 1). In the lower gamma range, the individuals with CP had greater power in the left (CP = 0.065 ± 0.025, NT = 0.037 ± 0.017, *p_FWE_* < 0.001) and right SII (CP = 0.040 ± 0.019, NT = 0.025 ± 0.012, *p_FWE_* = 0.001; Fig. 2). Similarly, the individuals with CP had greater power within the upper gamma range in the same areas of the left (CP = 0.052 ± 0.024, NT = 0.027 ± 0.014, *p_FWE_* < 0.001) and right SII (CP = 0.043 ± 0.017, NT = 0.027 ± 0.013, *p_FWE_* = 0.001) areas.

### Regression analysis

**Figure 3:**
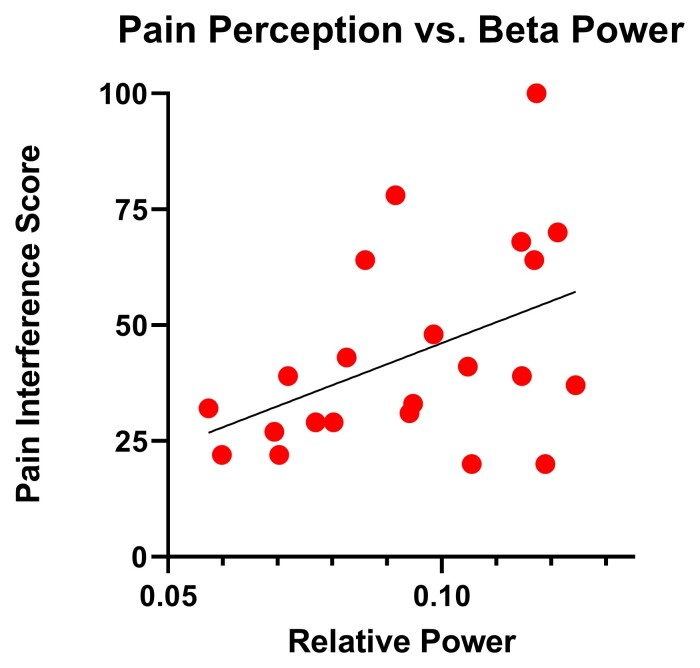
**Pain interference is associated with beta band activity**. Scores from the Pediatric Pain Interference Scale were predicted by the beta (15–29 Hz) power within the left SII cortex for the individuals with cerebral palsy. Individuals that had higher spontaneous beta power in the left SII also reported having more pain with their daily living (*P* = 0.039).

In the first block of the regression model, Pediatric Pain Interference scores were regressed on the spontaneous beta power within SII. The spontaneous beta power within SII significantly predicted the Pediatric Pain Interference scores (*F*(1,20) = 4.89, *P* = 0.039; Fig. 3). This implies that the individuals with CP that had stronger beta spontaneous activity also tended to report increased perception of pain affecting their daily living. No other frequency bands significantly predicted Pain Inference scores (*p*’s > 0.05).

Next, GMFCS level was added to the model to determine whether severity of impairment predicted pain interference. Thus, Pediatric Pain Interference scores were regressed on the spontaneous beta power within SII and GMFCS scores. With the addition of this predictor, the model was no longer significant (*F*(3,18) = 2.34, *P* = 0.108). Furthermore, GMFCS was not a significant predictor of pain interference (*P* = 0.179), and the interaction between GMFCS and spontaneous beta power was not significant (*P* = 0.213). These results are depicted in Table 1.

**Table 1:** Hierarchical regression model

Model	*b*	*SE*	*t*	β	*F*	*R* ^2^	Δ*F*	Δ*R*^2^	95% CI
1. Intercept	0.73	19.78	0.04		4.89*	0.20			[−40.54, 41.99]
SII Beta	453.58	205.15	2.21*	0.44					[25.64, 881.52]
2. Intercept	−68.31	53.78	−1.27		2.34	0.28	1.05	0.08	[−181.30, 44.67]
SII Beta	1119.42	571.33	1.96	1.09					[−80.91, 2319.75]
GMFCS	30.12	21.51	1.40	1.34					[−15.08, 75.31]
SII Beta X GMFCS	−288.67	223.81	−1.29						[−758.87, 181.53]

* *P* < 0.05.

## Discussion

In the present study, we utilized MEG brain imaging to quantify the spontaneous cortical activity of individuals with spastic diplegic CP and determined how alterations in such neural activity may be associated with the increased pain perception reported in this clinical population. Our results are the first to show that there are several cortical areas in which the strength of spontaneous activity was altered in the individuals with CP in comparison to the NT controls. These results imply that the perinatal brain insults experienced by individuals with CP have a broad impact on the brain’s overall spontaneous cortical activity. We expanded on these novel findings by also identifying that the altered spontaneous beta activity within SII predicted the increased pain perceptions reported by the participants with CP. These results further support the notion that disruptions in the spontaneous cortical activity might impact how individuals with CP process pain-related sensory information. In the following sections, we further discuss the implications of these findings.

### SII is connected with heightened pain perception

Our results are the first to show a positive association between spontaneous beta band activity within SII and the daily pain levels reported by individuals with CP. Pain is a significant problem that persists in many individuals with CP,^12–17,24^ yet the underlying neurophysiological mechanisms are not completely understood. Our findings point towards a specific neurophysiological parameter that may underlie this phenomenon. This finding corroborates previous literature that has identified heightened resting beta band activity in patient populations with chronic pain in many pain processing regions, including SII.^64–68^ In fact, numerous animal and human studies have exemplified that SII receives nociceptive input from the spinothalamic tract,^28,80–84^ indicating that it is a critical area for pain processing. Thus, the increased spontaneous activity within this area may be indicative of heightened sensitivity to the incoming pain signals ascending from the nociceptors. Overall, these data strongly support the idea that the heightened pain experienced by those with CP is not solely a product of abnormalities within the musculoskeletal system, but instead may have a prominent neural basis that is closely related to accentuated spontaneous beta activity in the SII cortex. Importantly, the results from our hierarchical regression analysis suggested that this relationship was not significantly modulated by impairment severity. This further supports the premise of a neurological component of pain rather than simply sequelae of a more severely affected musculoskeletal system.

### Altered spontaneous cortical activity in CP

The individuals with CP also exhibited altered spontaneous alpha and delta band activity within their occipital cortices. This is in line with our previous work that has demonstrated alterations in occipital alpha activity in CP while performing a visuomotor target matching task and processing visual stimuli.^59–61,85^ Potentially, the altered spontaneous activity may represent fundamental deficiencies in the ability of the visual cortices to process incoming information from the optic tract, thereby contributing to the cortical visual processing deficits. Multiple studies have linked spontaneous cortical activity levels to the neural oscillations that serve sensory and cognitive processing.^50,54,86–88^ The individuals with CP also exhibited decreased alpha band spontaneous activity within the left prefrontal cortex. This aligns with our previous work that has demonstrated that the alpha-beta oscillatory activity during the encoding phase of working memory is weaker in individuals with CP within the prefrontal cortex.^63^ Perhaps, the altered alpha activity in the prefrontal cortices could also be an indicator of the underlying neuropathology that is representative of the cognitive and intellectual deficits that are prominent and now widely recognized in this population.^1,89,90^

Another notable result from the present study was that the individuals with CP demonstrated increased spontaneous cortical activity within the beta and gamma bands within SII. Many studies have identified that the somatosensory cortical activity is aberrant in individuals with CP.^39,55,91–98^ Thus, the altered spontaneous activity within the SII may play a role in the disruption of incoming sensory information processing, resulting in the well-documented sensory impairments seen in the clinic.^5–7^ Alternatively, the increased activity within the beta band could be indicative of increased GABAergic activity. Prior pharmaco-MEG studies have shown that beta power is increased when a GABA_A_ receptor agonist is administered,^99^ as well as when a GABA transporter is blocked.^100^ Thus, increases in GABAergic activity intrinsically plays a role in the strength of spontaneous beta activity in the sensorimotor cortices. As such, the increased strength of the beta spontaneous activity may be reflective of heightened activity within the underlying GABAergic inhibitory interneurons. This view aligns with the prior PET studies that have shown that individuals with CP tend to have increased GABA_A_ receptor binding potential within the motor cortices.^101^

### Spontaneous cortical activity and oscillatory responses

As mentioned above, there is a growing consensus that the strength of the spontaneous oscillatory activity impacts the amount of change in the cortical oscillations that are induced by incoming stimuli or volitional movement.^50–54,86–88,102^ For example, it has been shown that the spontaneous sensorimotor beta oscillations are elevated in healthy ageing participants, and that these participants exhibit a larger beta desynchronization when producing a motor action.^52,53^ This implies that the sensorimotor cortices may have an absolute threshold that must be reached to produce a motor action. Along a similar line, other experimental outcomes have also identified that the somatosensory spontaneous cortical oscillations in the gamma frequency range are elevated in persons infected with HIV, and the relative change in gamma cortical oscillations is weaker following a peripheral stimulation.^103^ Similar findings have also been reported in the occipital cortices of persons with HIV.^102^ It is plausible that the altered spontaneous activity identified in this investigation might also play a role in the uncharacteristic cortical oscillations seen across the visual processing, working memory, somatosensory and motor domains in individuals with CP.^55,56,59–61,63,91,104^ Essentially, our current experimental outcomes provide the foundation for assessing this new hypothesis.

### Limitations

Before concluding, it is important to highlight some limitations within our study. First, our patient population only included individuals with spastic diplegic CP, limiting its generalization across other types (ataxic, athetoid, mixed) and presentations (hemiplegic, quadriplegic). Future investigations should determine whether changes in the resting-state activity in individuals with CP are ubiquitous across the entire population. Furthermore, our analysis was restricted to spontaneous cortical activity, without consideration of subcortical brain areas that may also exhibit altered resting-state activity. Investigating spontaneous activity in subcortical areas may further assist in understanding the altered neurophysiology that contributes to the sensorimotor and cognitive deficits, as well as the increased pain perception, within this population.

Furthermore, it should be noted that the heterogeneity in structural brain damage observed in those with CP could have potentially affected our estimates of spontaneous cortical activity. However, as our analysis was cortically constrained, and cortical lesions are not as common as other abnormal MRI findings in individuals with CP,^105,106^ this phenomenon likely had a negligible effect on our findings. In this context, it should also be noted that our sample consisted solely of individuals with spastic diplegic CP, and thus, hemispheric asymmetries in lesion impact were unlikely. Furthermore, some individuals with CP have no abnormal findings on their MRI,^38,107–109^ indicating that the relationship between structural lesions and functional brain aberrations in CP is complex and not fully understood. Despite these limitations, our findings suggest critical alterations in the spontaneous cortical physiology of individuals with CP that may be contributing to the range of impairments notable within this population.

## Conclusion

In conclusion, this study has provided clear evidence that individuals with CP display altered spontaneous cortical activity across multiple frequency bands and brain regions. Furthermore, individuals that have more strongly altered spontaneous activity within SII also tend to report increased pain that interferes with their daily life. Overall, these findings provide unique insight into the underlying pathophysiological mechanisms that contribute to the clinical impairments experienced in this population, and strongly support the notion that the initial brain insult results in adverse effects amidst several systems throughout the brain. Finally, these findings highlight the viability of MEG imaging as a tool in studying resting-state activity in individuals with CP. Potentially, changes in spontaneous resting-state activity may be utilized to measure the effectiveness of treatment strategies that are directed at reducing the pain seen in individuals with CP.

## Abbreviations

CP =: cerebral palsy
FC =: functional connectivity
FWE =: family-wise error
FWHM =: full width half maximum
GMFCS =: gross motor functional classification scale
MEG =: magnetoencephalography
MNI =: Montreal Neurological Institute
MRI =: magnetic resonance imaging
NT =: neurotypical
PET =: positron emission tomography
PSD =: power spectrum density
SII =: secondary somatosensory cortices
SSP =: signal-space projection
TFCE =: threshold-free cluster extraction
